# Age and race/ethnicity differences in decisional conflict in women diagnosed with ductal carcinoma in situ

**DOI:** 10.1186/s12905-024-02935-1

**Published:** 2024-02-04

**Authors:** Cecilia Portugal, Albert J. Farias, Erika L. Estrada, Aniket A. Kawatkar

**Affiliations:** 1 Department of Research and Evaluation, Kaiser Permanente Southern California, 100 So. Los Robles, Second Floor, Pasadena, CA 91101 USA; 2 Department of Preventative Medicine, Keck School of Medicine of USC, 2001 N. Soto Street Health Sciences Campus, Los Angeles, CA 90032 USA

**Keywords:** Decisional conflict, Ductal carcinoma in situ, Race/ethnicity

## Abstract

**Purpose:**

Women diagnosed with ductal carcinoma in situ (DCIS) face confusion and uncertainty about treatment options. The objective of this study was to determine whether there are differences in decisional conflict about treatment by age and race/ethnicity.

**Methods:**

A cross-sectional survey was conducted of women (age ≥ 18) diagnosed with DCIS enrolled at Kaiser Permanente of Southern California. The Decisional Conflict Scale (DCS) measured personal perceptions of decision uncertainty, values clarity, and effective decision-making. We used a multivariable regression to study whether age, race, and ethnicity were associated with patient-reported DCS.

**Results:**

45% (*N* = 1395) of women who received the online survey, participated. The mean age was 56 (± 9.6) years, the majority were white. Compared to women younger than 50, women aged 60–69 reported lower overall DCS scores (-5.4; 95% CI -1.5 to -9.3). Women > 70 had lower values clarity scores (-9.0; 95% CI -2.8 to -15.2) about their treatment compared to women aged 50–59 and 60–69 (-7.1; 95% CI -2.9 to -11.3 and − 7.2; 95% CI -2.9 to -11.5) and likewise, lower effective decision-making scores (-5.4; 95% CI -1.7 to -9.2 and − 5.2; 95% CI -1.4 to -9.0) compared to women < 50. Compared to whites, blacks reported lower decision conflict (-4.4; 95% CI 0.04 to -8.8) and lower informed decision (-5.2; 95% CI -0.18 to -10.3) about DCIS treatment.

**Conclusion:**

Younger women reported higher decisional conflict about DCIS treatment, compared to older women (> 70). Age based tailored discussions about treatment options, health education, and supportive decision-making interventions/tools may reduce decision conflict in future DCIS patients.

**Trade registration:**

The IRB number is 10678.

## Plain English summary

In the USA, approximately 50,000 women are diagnosed with ductal carcinoma in situ (DCIS) each year. Women diagnosed with DCIS experience confusion, uncertainty and have many questions about available treatment options. Patient’s confusion and uncertainty regarding DCIS, maybe related to receiving conflicting information and fear about developing invasive breast cancer after treatment. Our study findings help better understand the needs of women diagnosed with DCIS. The data we report from the study will help inform future studies and improve the body of knowledge in the management of DCIS. Our study quantifies the conflict experienced by women diagnosed with DCIS using the Decisional Conflict Scale. Our study found that younger women with DCIS reported greater difficulty with deciding about their DCIS treatment compared to older women with DCIS. These results are interesting because other studies have reported that older women have less factual knowledge, less education, and more health problems to consider when deciding about treatment for DCIS. This suggests a need for age-based tailored discussions about treatment options, health education, and supportive decision-making interventions/tools.

## Introduction

Ductal carcinoma in situ (DCIS) is stage zero non-invasive breast cancer and is believed to be a precursor of invasive breast cancer; DCIS makes up the majority of non-invasive cases however, there is no way to determine which cases will progress [1–3]. According to the American Cancer Society, in 2022, it was estimated that 51,400 women would be diagnosed with DCIS [4]. In a cohort study that utilized SEER data for women diagnosed with DCIS between 1995 and 2014, researchers found that the mean (SD) age of diagnosis was 57.4 [11.0] years and they had a 3-fold increased risk of death from breast cancer after surgical treatment [5]. The trends in DCIS incidence between 1998 and 2014 remained stable for white women but increased in African American, Hispanic and Asian-Pacific Islander women with the incidence rate being higher for African American and Asian-Pacific Islander women [6, 7]. The risk factors for DCIS are similar to invasive breast cancer and are associated with older age, and a family history of breast cancer.

Decisional conflict regarding DCIS is often due to no previous awareness of the disease and a lack of understanding of treatment options [8]. A diagnosis of DCIS can result in confusion among patients due to lack of knowledge about the disease, treatment options, and how it differs from invasive breast cancer [9–13]. Despite the fact that women diagnosed with DCIS have similar treatment options compared to early-stage invasive breast cancer which can include mastectomy, breast conserving surgery (BCS) and/or radiation with or without hormone therapy [13–16], there is a gap in the field surrounding decisional conflict about treatment for DCIS among older women as very few studies have addressed this issue. A recent study found that women in the 70–74 year groups believed that if they were older than they currently were, they would not worry about DCIS and might be more comfortable with monitoring DCIS [8]. Patients often rely on their health care providers to guide them through their diagnosis and treatment plan, but studies have shown that there is a need for improved communication between patients and providers to help patients make more informed decisions about treatment [12, 17]. There is also controversy about overtreatment of DCIS however, research has shown that 20–50% of patients with untreated DCIS later advance to invasive disease and when a decision has been made to treat, women want to know more about the difference between conservative treatment vs. mastectomy [18, 19].

Studies have focused on patient understanding of treatment for DCIS and found differences by age and race/ethnicity. For instance, younger women have more concerns about developing breast cancer, the disease metastasizing, and rate their risk for invasive cancer higher than older women [10]. A general lack of awareness among women diagnosed with DCIS has been shown to result in decisional conflict about understanding the disease and the available treatment options. One study suggests that women are confused and misunderstand their diagnosis of DCIS and 60% of those surveyed thought DCIS could metastasize [9]. A recent mixed-methods study found that women who were treated for DCIS did not all have a clear understanding of DCIS which contributed to their confusion [20]. Studies have reported there are racial/ethnic and age-related differences in treatment knowledge among women diagnosed with DCIS which could influence decisional conflict about treatment [21].

The objective of this study was to evaluate the association between age, race/ethnicity, and decisional conflict regarding treatment among patients diagnosed with DCIS.

## Methods

### Study setting

Founded in 1945, Kaiser Permanente is one of the nation’s largest not-for-profit integrated healthcare systems in which hospitals, health plans, and medical groups collaboratively operate in over nine states and Washington D.C. The Kaiser Permanente Southern California (KPSC) Medical Care Program is a large integrated care organization with over 4 million members. Members receive their health care throughout the seven-county region in which KPSC has 14 medical centers and affiliated hospitals, along with 214 medical offices. The membership represents over 260 different ethnicities that speak about 115 different languages. Members enroll through the Kaiser Foundation Health Plan for pre-paid healthcare insurance, including pharmacy benefits. Health care at KPSC is coordinated through region-wide electronic medical records (EMR) that capture detailed information on care provided to members at outpatient visits, inpatient stays, as well as utilization of pharmacy, immunizations, imaging, and laboratory services. Patient demographic information, including date of birth, sex, race/ethnicity, and health plan enrollment information, were obtained from KPSC membership enrollment. In this study, the research involving human participants, human material, or human data, have been performed in accordance with the Declaration of Helsinki and have been approved by the Institutional Review Board for Kaiser Permanente Southern California which is the ethics committee charged with protecting the rights and welfare of people involved in research at Kaiser Permanente Southern California.

### Criteria for study inclusion

Patients, ages 18–100 years were included if they had a history of DCIS (stage 0 breast cancer) documented in the KPSC Cancer Registry. Patients were excluded if they did not have an email address identified in the HealthConnect^®^ system of KPSC. Patients who were cognitively impaired were not included in the study. Surveys were conducted in English.

### Survey administration

A survey questionnaire was developed using validated scales to assess patient reported outcomes among KPSC members diagnosed with DCIS. The Decisional Conflict Scale (DCS) was validated and used to measure personal perceptions of decision uncertainty, values clarity, and effective decision-making [22]. The survey was implemented using DatStat (datstat.com). Participants were sent an encrypted email invitation and introductory letter with a Frequently Asked Questions sheet that contained informed consent language, a link to opt-out of the study if they no longer wanted to receive correspondence about the study or were not interested, and an encrypted link to complete the survey online. Email reminders were sent for non-respondents: three days after the first email and at the beginning of the second and third week. Finally, follow-up telephone calls for non-respondents were conducted at the beginning of week 3. The survey concluded approximately 1-month after the introductory email was sent. There was no compensation for study participation. The survey took 25–30 min to complete.

### Decisional conflict scale

We assessed the five domains in the traditional Decisional Conflict Scale (DCS) to measure personal perceptions of patient treatment for DCIS. Scores for each domain and overall DCS range from 0 to 100 with higher scores indicating higher decisional conflict. The DCS scale has been described in detail elsewhere [22]. Briefly, the five domains are the following: 1) Informed subscale: feeling uninformed about treatment. The scale asks questions about “Knowing what options are available, the benefits of options and risks and side effects of treatment options.” 2) Values clarity subscale: feeling unclear about personal values. The scale asks questions about “Clear about what benefits matter most to the individual, which risk and side effects matter most and what is most important.” 3) Support subscale: feeling unsupported in treatment decision making. The scale asks questions about “Feeling that they have enough support from others to make the choice; making a choice without feeling pressure from others and; feeling they had enough advice to make a choice.” 4) Uncertainty subscale: feeling uncertainty in choosing options. The scale asks questions about “Feeling they have made the best choice for themselves; feeling sure about the treatment that they chose, and that the decision was easy to make.” 5) Effective decision subscale: feeling the treatment choice is informed, value-based, likely to be implemented, and satisfaction with choice. The scale asks questions about “Feeling they made an informed choice; the decision they made shows what is important to them and do they expect to stick to the decision.”

### Covariates

Socio-demographic and clinical factors that could confound the association of decision conflict with exposures of interest were identified from literature and abstracted from the EMR and survey data. The socio-demographic covariates included age, (< 50, 50–59, 60–69 and ≥ 70); race (white, African American, Asian, Pacific Islander, American Indian, Alaskan Native) and ethnicity (Hispanic, non-Hispanic). Clinical factors included Estrogen receptor status (ERA positive/negative); Progesterone receptor status (PRA positive/negative) and comedocarcinoma type DCIS (the high-grade subtype of DCIS). We also included a history of cancer (yes/no), educational attainment, the log of area median income, and the Charlson comorbidities (0, 1, 2, and ≥ 3) as covariates. Household make-up for each participant was coded as living alone, living with spouse (or life partner), living with adult children, living with other adults who are able to help, and living with children under the age of 18.

### Statistical analysis

Descriptive statistics including means, medians and proportions were used as appropriate to describe the socio-demographic and clinical characteristics as well as the range of the survey domain scores. To evaluate the association in decisional conflict about DCIS treatment in women of varying age and race/ethnicity, we used multivariable regression models with age, race, and ethnicity as exposure variables, controlling for other demographic and tumor characteristics. Separate regression models were evaluated for the subscales as well as a combined decisional conflict score. Hypothesis testing was based on heteroscedasticity-consistent standard errors of the regression models to avoid misspecification bias. We also evaluated if the model met the regression assumptions requirements.

## Results

### Sample characteristics

A total of 3,092 women met the criteria for study inclusion of which 1,395 submitted a completed/partially completed survey which resulted in a response rate of 45.1%. Of the 1395 women who participated in the survey, 989 completed or partially completed the Decisional Conflict Scale and the data presented henceforth will reflect the results of these 989 participants. Their mean age at diagnosis was 56.2 (± 9.5) and the majority were white (73.3%) followed by Hispanic (14.1%), Asian (10.2%), Black (10.1%) and other race (6.3%) (Table 1). Slightly over half (52.3%) of the study participants reported an annual income that ranged from $65,001 to greater than $150,000 and a majority of the study participants had a spouse/life partner (45.0%) while 54.3% of the women in our cohort lived with either a spouse/partner/adult children/or other adults who were able to help. Over one-quarter of the sample (28.3%) had some college education/no degree and 59.7% had an associate degree, a college degree or completed graduate school/professional degree (Table 1).

**Table 1:** Descriptive statistics

Number of patients (n)	N = 989
**Age Mean (SD)**	
Age at DCIS diagnosis	56.2 (9.5)
**Race N (%)**	
White	725 (73.3%)
Black	100 (10.1%)
Asian	101 (10.2%)
Other/Unknown/Multiple	63 (6.4%)
**Ethnicity N (%)**	
Hispanic	139 (14.1%)
Non-Hispanic	850 (85.9%)
**Income Mean (SD)**	
Under $35,000	81 (8.2%)
$35,001 - $65,000	210 (21.2%)
$65,001 – $100,000	231 (23.4%)
$100,001 – more than $150,000	286 (28.9%)
Unknown/missing	181(18.30%)
**Education N (%)**	
Did not graduate from high school	15 (1.5%)
High school graduate or equivalent	102 (10.3%)
Some college, no degree	280 (28.3%)
Associate college degree	121 (12.2%)
4-year college degree	197 (19.9%)
Graduate or professional degree	273 (27.6%)
Unknown/missing	1(0.1%)
**Lives in household N (%)**	
I live alone	175 (17.7%)
Spouse (or life-partner)	445 (45.0%)
Adult children	62 (6.3%)
Other adults who are able to help	30 (3.0%)
Children under age 18	13 (1.3%)
Other (household members)	152 (15.4%)
Missing/Unknown	112 (11.3%)
**ERA Positive N (%)**	
Yes	483 (48.8%)
No	506 (51.2%)
**PRA Positive N (%)**	
Yes	332 (33.6%)
No	657 (66.4%)
**Comedo type DCIS N (%)**	
Yes	96 (9.7%)
No	893 (90.3%)
**High Grade Tumor Cells N (%)**	
Yes	151 (15.3%)
No	838 (84.7%)
**Had Personal Cancer History N (%)**	
Yes	226 (22.9%)
No	763 (77.1%)

The women diagnosed at age ≥ 70 had the lowest average overall DCS score [mean: 16.84, SD:(20.72)] compared to the other age groups and women < 50 had the highest average overall DCS score [21.48 (22.48)] (Table 2). In terms of race, African Americans had the lowest average overall DCS score [14.92(17.15)] while Alaskan Natives, Native Americans/ Multiples, other races had the highest average overall DCS score among the racial groups [23.19 (24.68)]. Hispanics had a higher average DCS score [21.00 (23.31)] compared to non-Hispanics [18.33 (20.86)].

**Table 2:** Mean decisional conflict (DC) Overall and subscale scores by race, ethnicity, and age

	Overall DC Score (Range 0-100)	Uncertainty subscale score (Range 0-100)	Informed subscale score (Range 0-100)	Values clarity subscale score (Range 0-100)	Support subscale score (Range 0-100)	Effective decision subscale (Range 0-100)
	Mean (SD)	Mean (SD)	Mean (SD)	Mean (SD)	Mean (SD)	Mean (SD)
**Total Cohort**	18.71 (21.08)	21.80 (23.45)	20.00 (24.99)	19.65 (24.47)	17.96 (22.48)	16.65 (21.18)
**Age at diagnosis**						
< 50	21.48 (22.48)	25.57 (25.10)	21.65 (26.13)	22.04 (25.66)	21.13 (24.08)	19.48 (22.86)
50–59	18.07 (21.54)	20.79 (23.74)	19.50 (25.38)	19.11 (24.62)	17.37 (22.09)	15.40 (20.88
60–69	17.55 (19.28)	19.82 (21.34)	19.22 (23.78)	19.19 (24.00)	16.97 (21.58)	15.29 (18.99)
≥ 70	16.84 (20.72)	22.22 (23.79)	20.15 (24.37)	15.89 (21.01)	17.68 (22.32)	18.66 (24.64)
**Race**						
White	18.89 (20.87)	22.23 (23.37)	20.29 (25.02)	19.83 (24.55)	17.96 (22.44)	16.81 (21.23)
African American	15.93 (17.15)	18.41 (20.47)	16.74 (21.30)	18.75 (21.53)	15.33 (18.51)	14.19 (16.75)
Asian	17.39 (23.37)	19.53 (25.03)	17.47 (24.01)	16.59 (24.43)	21.82 (25.29)	15.93 (23.79)
Other/Unknown/Multiple	23.19 (24.68)	25.78 (25.76)	26.15 (30.47)	23.97 (27.81)	20.10 (23.74)	20.00 (22.73)
**Ethnicity**						
Hispanic	21.00 (22.31)	25.46 (23.93)	22.76 (26.81)	21.47 (25.54)	20.10 (23.74)	18.36 (21.31)
Non-Hispanic	18.33 (20.86)	21.21 (23.33)	19.56 (24.67)	19.36 (24.30)	17.62 (22.26)	16.37 (21.16)

In the multivariable analysis (Table 3), compared to women < 50, women 60–69 reported lower overall DCS score (-5.4; 95% CI -1.5 to -9.3). Women > 70 had lower values clarity score (-9.0; 95% CI -2.8 to -15.2) compared to other age groups. Women aged 50–59 and 60–69 reported similarly lower decision uncertainty scores about their treatment (-7.1; 95% CI -2.9 to -11.3 and − 7.2; 95% CI -2.9 to -11.5) and likewise, lower effective decision-making scores (-5.4; 95% CI -1.7 to -9.2 and − 5.2; 95% CI -1.4 to -9.0) compared to women < 50. Women aged 50–59 scored the lowest on the support subscale compared to the other age groups (-4.5; 95% CI -0.57 to -8.6). Compared to Whites, African Americans reported lower decision conflict about their DCIS treatment (-4.4; 95% CI -0.04 to -8.8) and lower informed decision (-5.2; 95% CI -0.18 to -10.3). We did not observe any decisional conflict score differences between Hispanics vs. non-Hispanics.

**Table 3:** Multivariable analysis evaluating differences in decisional conflict (DC) and subscale scores by age, race, and ethnicity (n = 989)

	Overall DC Score	Uncertainty subscale score	Informed subscale score	Values clarity subscale score	Support subscale score	Effective decision subscale
	(95% CI)	(95% CI)	(95% CI)	(95% CI)	(95% CI)	(95% CI)
**Age at diagnosis**						
< 50	-	-	-	-	-	-
50–59	-5.04 (-8.86, -1.22)	-7.14 (-11.35, -2.93)	-4.29 (-8.73, 0.14)	-5.35 (-9.77, -0.93)	-4.59 (-8.62, -0.57)	-5.46 (-9.20, -1.72)
60–69	-5.43 (-9.30, -1.56)	-7.22 (-11.54, -2.90)	-3.78 (-8.38, 0.81)	-4.87 (-9.42, − 0.32)	-4.14 (-8.38, 0.10)	-5.25 (-9.07, -1.43)
≥ 70	-6.94 (-12.86, -1.01)	-6.96 (-13.13, -0.79)	-5.91 (-12.5, 0.68)	-9.05 (-15.23, -2.87)	-6.23 (-11.99, -0.46)	-5.29 (-11.29, 0.70)
**Race**						
White	-	-	-	-	-	-
African American	-4.40 (-8.85, 0.044)	-4.54 (-9.59, 0.51)	-5.26 (-10.34, -0.18)	-2.83 (-7.95, 2.29)	-3.69 (-8.28, 0.90)	-2.92 (-7.25, 1.39)
Asian	-1.21 (-6.51, 4.09)	-3.11 (-8.81, 2.57)	-1.70 (-7.19, 3.79)	-2.11 (-7.78, 3.55)	0.84 (-4.70, 6.38)	-0.11 (-5.36, 5.13)
Other/Unknown/Multiple	3.69 (-2.87, 10.25)	1.49 (-5.36, 8.34)	6.07 (-2.11, 14.26)	5.04 (-2.81, 12.90)	2.74 (-4.25, 9.74)	2.58 (-3.42, 8.58)
**Ethnicity**						
Non-Hispanic	-	-	-	-	-	-
Hispanic	-2.18 (-6.14, 1.76)	0.04 (-4.36, 4.45)	-2.58 (-7.63, 2.46)	-3.86 (-8.74, 1.01)	-1.44 (-6.04, 3.15)	-1.84 (-5.57, 1.87)

*Additional covariates adjusted were income, Charlson comorbidity, clinical factors included Estrogen receptor status (ERA positive/negative); Progesterone receptor status (PRA positive/negative) and Comedo type DCIS, family history of cancer, educational attainment, the log of area median income

## Discussion

In this large study of women diagnosed with DCIS we combined data from a survey of self-reported decisional conflict with electronic health records of a large managed care organization and found that younger women with DCIS reported significantly higher decisional conflict regarding their DCIS treatment compared to older women with DCIS. These results are interesting because other studies have reported that older women have less factual knowledge, less education, and more comorbidities to consider when making a decision about treatment for DCIS as age increases [23].

Decisional conflict among older women diagnosed with breast cancer has been shown to be related to factors such as comorbid health problems, family responsibilities, caregiving and family/personal history with breast cancer [24]. Bleicher et al. sought to understand the role of age and how age-related factors play a role in surgical decision-making. The authors found that younger women (< 41) had the highest rates of mastectomy but overall, surgical decision did not vary significantly by age [23]. They also found that 40% of the women > 70 had more involvement in their decision making than they would have preferred and that older women had less factual knowledge about their choice [23]. Treatment decisions for older women can be complicated by other existing health problems, life expectancy, and knowledge and understanding of the disease. A study by the National Comprehensive Cancer Network suggests that treatment not be based on age alone but on the patient’s goals, life expectancy and the totality of comorbidities [25]. Patient decisional conflicts can be addressed with the help of new technology (Oncotype DX® DCIS Score) which can be utilized to inform clinicians in their assessment for adjuvant therapy options as well as helping patients make more informed therapeutic decisions about their care and future risk assessment of breast cancer [26–29].

Overall, one reason older women in our study may have experienced less decisional conflict when compared to younger women is that they may have had more social support from family members (e.g. adult children) helping them make informed decisions. Over 50% of the women in our cohort lived with either a spouse/partner/adult child or another adult who was able to help, which may be indicative of having social support and help with making important health care decisions. Also, physicians play a large role in helping older women make more informed decisions about treatment. Schonberg et al. showed that physicians detailed the complex and individualized treatment plan for decision-making in a cohort of women aged ≥ 80 diagnosed with breast cancer, which helped those women make more informed decisions [24]. Ruddy et al. showed that the risk perception of DCIS recurrence within 5 years or within a lifetime, among women ≤ 54 years old, was greater than in older women and this may greatly influence decisional conflict among younger women [30]. Furthermore, several studies showed that younger women had higher decisional conflict regarding cancer treatment and specifically breast cancer treatment (e.g. surgical treatment) [31–33]. Also, younger women may feel uninformed about fertility preservation options and that they do not have enough advice or support to make decisions [31]. This may result in greater decisional conflict during cancer treatment for women who are not referred to fertility preservation [32].

Women could experience high decisional conflict if they do not understand their diagnosis, which is a particular issue for women diagnosed with DCIS. De Morgan et al. revealed that study participants did not understand how a diagnosis of DCIS was different than invasive breast cancer and were confused about whether they had “cancer” or not because of conflicting descriptions about DCIS amongst health care providers [9]. Our results imply that health care providers may need guidance on how to communicate the uncertainty of DCIS and the natural history of the disease more effectively to younger women since they had more decisional conflict compared to older women. Nevertheless, it is critical that women at every age (young and older) be informed and educated about DCIS and how it differs from invasive or metastatic breast cancer and what are the treatment options and risks involved.

In our study, we observed that African Americans had a lower decisional conflict score than whites. A lower DCS score indicates that the women were more confident about their treatment decision, had knowledge about the disease/treatment, and were clear about what treatment choice was good for them. This result is contrary to what the literature shows for African Americans in that they have greater distrust of the medical care system compared to other groups, receive less aggressive treatment when compared to whites, and have worse outcomes [34, 35]. Studies have shown that African Americans have a lower chance of 5-year cancer survival than non-Hispanic Whites [36, 37]. A few studies showed that African Americans had a higher risk of death from DCIS than non-Hispanic whites and this was also true for African Americans when compared to other ethnic groups due to the development of aggressive breast cancer following DCIS [37–39]. These findings should be considered when making treatment decisions for African American women with DCIS.

This is one of the largest studies to link a survey of self-reported decisional conflict to electronic health records controlling for clinical and demographic characteristics which was possible because the patient population at this large health plan is stable and are typically long-standing patients with the health plan. However, there are limitations. First, while the survey data were cross-sectional and may not capture changes in DCS score over time, in a sensitivity analysis we included a variable measuring the time from diagnosis to survey administration and it was not significantly associated with DCS scores. Second, the study time frame included women diagnosed with DCIS over a 16-year time period (1998 to 2014) thus there may be some recall bias because we relied on memory and patient recall/self-report about their DCIS diagnosis and treatment experience. Finally, we relied on patients to read their emailed invitation letter and participate in the survey with few reminders and no stipend offer; this could have negatively impacted our response rates making it more likely that the response rate is a reflection of survey logistics rather than experiences with breast cancer.

## Conclusion

The results from this study suggest that more work needs to be done to aid all women of all ages diagnosed with DCIS in making patient-centered treatment decisions. This study highlights that patient age is an important factor to consider because they have different needs for making decisions for different phases in the life and during the aging process. The focus should be on educating women about how DCIS differs from invasive breast cancer. Additionally, research is needed to determine if older women have more knowledge about DICS or if they are receiving greater physician recommendations and family involvement in their decision-making regarding DCIS treatment compared to younger women. To improve decisional conflict, a greater focus on patient-physician communication and patient involvement in decision making should include a discussion about the technical, risk assessment, benefits, and uncertainties about treatment. The use of decision aids (educational pamphlets, Web sites) help patients understand information about diagnosis and treatment options as well as help them express their personal values associated with treatment options which can significantly reduce patients’ decisional conflict regarding treatment [17, 20, 40]. A recent study identified how existing tools on the internet could be improved for patient communication and found higher quality tools that clinicians could use when discussing DCIS with their patients [41].

## Data Availability

Because of the sensitive nature of the data collected for this study and to protect health information, requests to access the dataset is not available. Most study material to construct the covariates are available within the article. Additional details are available from the corresponding author upon reasonable request.

## Abbreviations

DCIS: Ductal Carcinoma in Situ
DCS: Decisional Conflict Score
CI: Confidence Internal
